# Clinical Application of the Fluid Challenge Approach in Goal-Directed Fluid Therapy: What Can We Learn From Human Studies?

**DOI:** 10.3389/fvets.2021.701377

**Published:** 2021-08-03

**Authors:** Francisco José Teixeira-Neto, Alexander Valverde

**Affiliations:** ^1^Departmento de Cirurgia Veterinária e Reprodução Animal, Faculdade de Medicina Veterinária e Zootecnia, Universidade Estadual Paulista, Botucatu, Brazil; ^2^Department of Clinical Studies, Ontario Veterinary College, University of Guelph, Guelph, ON, Canada

**Keywords:** fluid challenge, fluid responsiveness, goal-directed fluid therapy, shock, cardiac output

## Abstract

Resuscitative fluid therapy aims to increase stroke volume (SV) and cardiac output (CO) and restore/improve tissue oxygen delivery in patients with circulatory failure. In individualized goal-directed fluid therapy (GDFT), fluids are titrated based on the assessment of responsiveness status (i.e., the ability of an individual to increase SV and CO in response to volume expansion). Fluid administration may increase venous return, SV and CO, but these effects may not be predictable in the clinical setting. The fluid challenge (FC) approach, which consists on the intravenous administration of small aliquots of fluids, over a relatively short period of time, to test if a patient has a preload reserve (i.e., the relative position on the Frank-Starling curve), has been used to guide fluid administration in critically ill humans. In responders to volume expansion (defined as individuals where SV or CO increases ≥10–15% from pre FC values), FC administration is repeated until the individual no longer presents a preload reserve (i.e., until increases in SV or CO are <10–15% from values preceding each FC) or until other signs of shock are resolved (e.g., hypotension). Even with the most recent technological developments, reliable and practical measurement of the response variable (SV or CO changes induced by a FC) has posed a challenge in GDFT. Among the methods used to evaluate fluid responsiveness in the human medical field, measurement of aortic flow velocity time integral by point-of-care echocardiography has been implemented as a surrogate of SV changes induced by a FC and seems a promising non-invasive tool to guide FC administration in animals with signs of circulatory failure. This narrative review discusses the development of GDFT based on the FC approach and the response variables used to assess fluid responsiveness status in humans and animals, aiming to open new perspectives on the application of this concept to the veterinary field.

## Introduction

Patients that are critically ill or that are undergoing major surgery are frequently presented with inadequate tissue oxygen delivery due to poor circulating volume. Under these conditions, rapid volume expansion *via* intravenous fluid administration should optimize tissue perfusion but such benefit is not without risks. Iatrogenic hypervolemia due to a liberal fluid administration strategy causes tissue/lung edema and worsens patient outcome (1). Otherwise, insufficient fluid administration due to a restrictive fluid therapy strategy can perpetuate hypovolemia/poor tissue perfusion and increase the rate of complications such as renal failure and death (1). According to guidelines published in veterinary medicine, when signs of circulatory failure/hypovolemia are present, aggressive fluid administration can be initiated *via* rapid administration of isotonic crystalloids using 25% of the “shock dose” (15–20 ml/kg in dogs and 10 mL/kg in cats), while monitoring the patient's the response (e.g., blood pressure, capillary refill time, lung sounds) (2). Based on the response to initial volume expansion, additional aliquots (25% of shock dose) can be administered, or a synthetic colloid can be considered if 50% of crystalloid shock volume did not cause sufficient improvement (2). However, there are opened questions regarding the goals and endpoints that should guide volume resuscitation in animals.

In the human medical field, fluid resuscitation guided by assessment of fluid responsiveness [i.e., the ability of an individual to increase stroke volume (SV) and cardiac output (CO) in response to volume expansion] appears to reduce patient mortality, duration of stay in the intensive care unit (ICU), and duration of mechanical ventilation (3). The use of a fluid challenge approach (i.e., the rapid administration of a relatively small amount of fluids) guided assessment of fluid responsiveness status allows rapid replacement of intravascular volume deficits as long as the individual has a cardiac preload reserve (i.e., is positioned on the ascending limb of the Frank-Starling curve). Because additional amounts of fluids are administered only if the individual presents an improvement in SV and CO, the fluid challenge approach can improve tissue perfusion and minimize the risks associated with hypervolemia and fluid overload (1, 3). This article will review the physiological basis of goal-directed fluid therapy (GDFT), the fluid challenge approach for treating circulatory failure, and the target variables used to evaluate the response to volume resuscitation. Most of the current literature originates from research on the human side, whether conducted in the clinical setting, or from bench research in animals. More recently, veterinary studies have used this information in several animal species. This article aims to critically review the development of the fluid challenge approach in human and veterinary medicine, with the objective of shedding light on the possible applications of this concept to the veterinary field.

References from PubMed database since January 2000 were included in this narrative review. Primary search terms were fluid challenge, GDFT, and fluid responsiveness. Human and animal studies (dogs and cats, and other species if relevant) involving the above mentioned terms were selected. To approach the target variables used to assess the response to a fluid challenge, studies comparing newer CO measurement technologies with reference methods of CO measurement in humans, dogs, and cats were selected. Studies published before 2000 considered seminal for understanding the current state of knowledge were also included in the review.

## The Physiological Basis of Goal Directed Fluid Therapy

**Figure 1:**
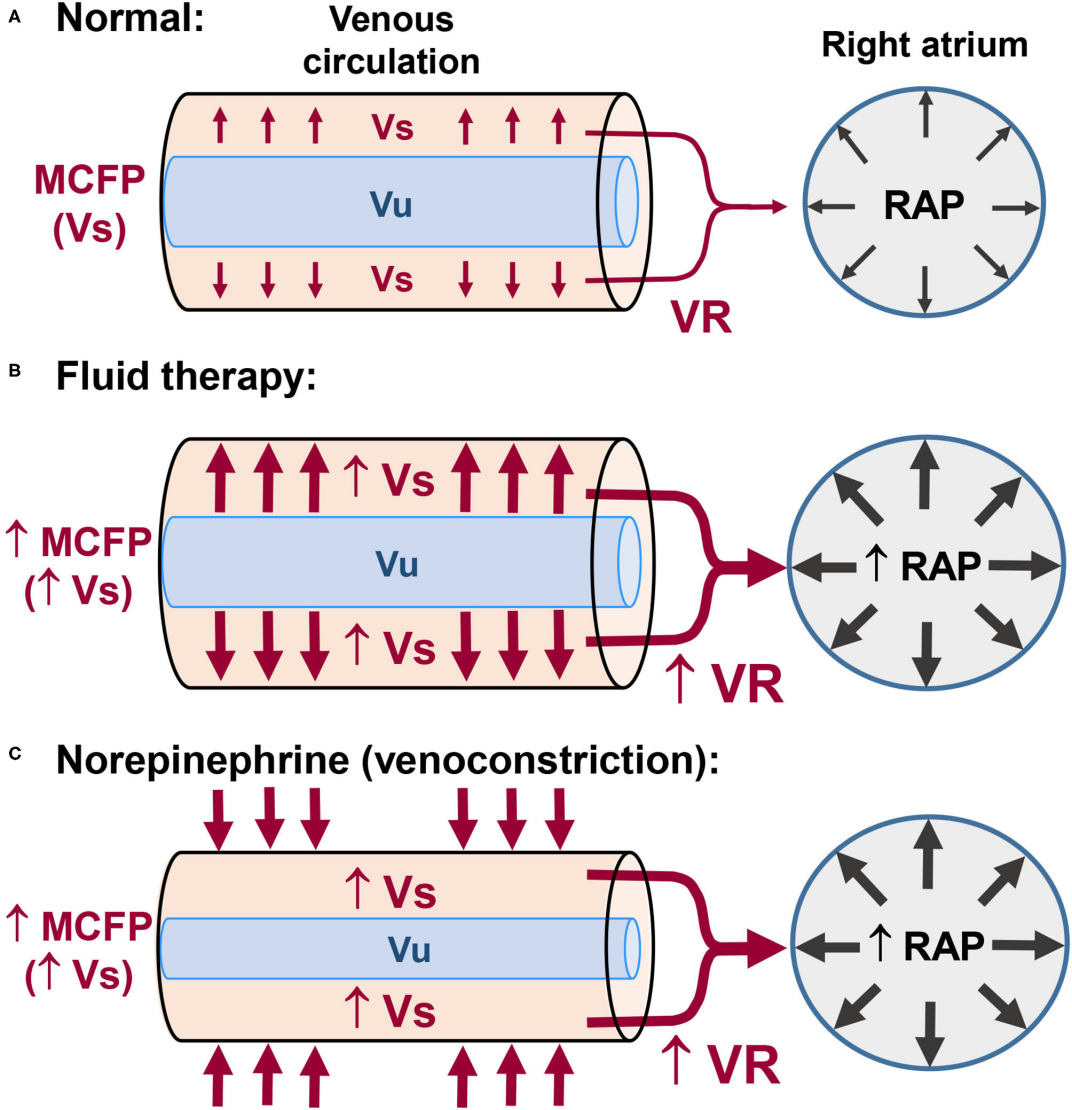
Control of venous return (VR) in normal subjects and the effects of fluid therapy and of vasopressors on VR. **(A)** The unstressed blood volume (Vu) maintains the vessels minimally opened. The stressed blood volume (Vs) exerts pressure on the vessel walls and is responsible for the mean circulatory filling pressure (MCFP). The pressure gradient between the upstream veins (MCFP) and right atrial pressure (RAP) is the major driving force responsible for VR of blood from the systemic circulation to the right side of the heart. **(B)** Intravenous fluid administration may increase VR and RAP (preload) *via* increases in Vs and MCFP. **(C)** Venoconstriction induced by low doses of vasopressors (e.g., norepinephrine) may also increase VR and RAP *via* increases in Vs and MCFP.

The venous system is composed of highly compliant capacitance vessels (i.e., vessels that can accommodate a large volume of blood without a large increase in intravascular pressure) which contain ~70% of blood volume (4, 5). Based on its physiological role in the circulation, blood volume is subdivided into unstressed and stressed volumes (Vu and Vs, respectively), which represent 70% and 30% of total blood volume, respectively (4, 5). The Vu is the blood volume that maintains the vessels minimally opened and does not influence transmural pressure (i.e., the difference between intravascular pressure and the pressure exerted on the outside of the vessel wall). The Vs is the blood volume that stretches the vessel walls, effectively increasing transmural pressure (4, 5). The Vs represents the blood that moves fast within the circulatory system and is the major determinant of venous return (VR) (4, 5). During steady state conditions, VR from the systemic and pulmonary circulations equals CO. Venous return is the product of the gradient between mean circulatory filling pressure (MCFP) and right atrial pressure (RAP), divided by the resistance to blood flow in the venous circulation (Figure 1A). The effects of a fluid challenge on VR, SV, and CO may be the result of an unpredictable distribution of the infused fluid between the Vs and Vu, which may explain why ~50% of critically ill human patients do not respond to a fluid challenge with significant increases in SV and/or CO (6). If intravenous fluids are distributed mainly to Vu there will be no increase in VR, SV, and CO (6). Otherwise, a fluid challenge will result in the desired increase in VR, SV, and CO if it augments MCFP *via* an effective increase Vs (Figure 1B). Another frequently unappreciated physiological concept is the fact that fluids do not hold the monopoly of improving VR, SV, and CO. Venoconstriction may also shift blood from Vu to Vs, thereby, increasing VR due to an increase in MCFP (Figure 1C). Lower doses of vasopressor agents (e.g., norepinephrine and phenylephrine) may increase VR, SV, and CO through a venoconstrictive effect (6, 7). Otherwise, higher doses of vasopressors, in spite of increasing VR and SV through venoconstriction, will also induce significant arterial vasoconstriction, which could eventually lead to a decrease in CO (6). The negative impact of vasopressors on blood flow/CO can be suspected if a substantial increase in arterial pressure induced by these drugs is paralleled by clinically relevant bradycardia, which is probably caused by activation of the baroreceptor reflex.

## What is Fluid Responsiveness and How Can it Be Assessed?

In conscious patients with signs of acute circulatory failure (e.g., hypotension, increased blood lactate levels), intravascular volume expansion is beneficial only if it significantly increases SV and CO. Titration of intravenous fluids to maximize SV and CO, while avoiding hyper- or hypovolemia is an ongoing paradigm in the medical field. The Frank-Starling curve provides the physiological basis for understanding the concept of fluid responsiveness. To test the fluid responsiveness status of an individual, a change in preload must be provoked and the observed response in SV or CO recorded (8). Responders to volume expansion, whose heart is operating in the ascending limb of the Frank-Starling curve, are defined as those individuals where SV or CO increase at least 10–15% after an increase in preload induced by test dose of fluids (i.e., a fluid challenge) or by a passive leg raise test (9–11). The passive leg raise test in humans involves raising both legs to a 45° angle in relation to the trunk to temporarily increase cardiac preload by diverting part of the blood volume (~300 ml) from the legs to the trunk/central circulation. This test allows determining the fluid responsiveness status of an individual without the need to administer fluids (11). In veterinary medicine, determination of fluid responsiveness has been restricted to the evaluation of changes in SV or CO in response to a fluid challenge in anesthetized or conscious dogs (12–22).

Dynamic assessment of fluid responsiveness relies on changes in preload induced by mechanical ventilation and the resulting change in surrogates of SV (23–29). Differently from the fluid challenge approach, which relies on a test dose of fluids to determine responsiveness status, heart/lung interactions during mechanical ventilation allows identifying the relative position of an individual in the Frank-Starling curve (i.e., fluid responsiveness status) *a priori* to fluid administration (23–29). Because preload changes induced by volume-controlled ventilation cause larger SV variations in patients positioned in the ascending limb of the Frank-Starling curve, responders to volume expansion can be identified if respiratory induced variations in SV are increased above a certain threshold. Surrogates of SV used for dynamic assessment of fluid responsiveness include respiratory variation in peak aortic flow velocity (ΔV_peak_), pulse pressure variation (PPV), systolic pressure variation (SPV). plethysmographic variability index (PVI) and stroke volume variation derived from pulse contour analysis (SVV_PCA_) (29).

In contrast with dynamic preload indexes, static markers of preload that reflect cardiac filling pressures (central venous pressure and pulmonary artery occlusion pressure), or that reflect end-diastolic volume, are not reliable to predict fluid responsiveness and have limited usefulness to guide fluid administration (30–32). Accurate prediction of fluid responsiveness by PPV and other dynamic preload indexes demands that patients are under volume-controlled mechanical ventilation with tidal volumes ≥8 ml/kg, without heart rhythm irregularities and without spontaneous breathing efforts (33). According to a meta-analysis, PPV was superior to predict responsiveness than other dynamic preload indexes in human patients under mechanical ventilation with tidal volumes ranging from 8 to 12 ml/kg (31). Although, PPV values >12.5% accurately discriminate responders from non-responders to a fluid challenge (31), this index has limited clinical application in the ICU setting because of inaccurate prediction of responsiveness in patients ventilated with low tidal volumes (<8 ml/kg) (34, 35). The ability of ΔV_peak_, PPV, SPV, PVI, and SVV_PCA_ to predict the response to a fluid challenge has been assessed in anesthetized mechanically ventilated dogs (12–16, 18, 21, 22). In canine studies PPV thresholds > 11–16% were able to identify responders to volume expansion (13–16, 18, 21, 22). The large variation in PPV thresholds reported by the veterinary literature can be explained in part by differences in the fluid challenge protocol, in the methods used for assessing the response to volume expansion, and in mechanical ventilation adjustments.

Although, dynamic preload indexes are effective tools to predict fluid responsiveness, the necessity for patients to be under mechanical ventilation without cardiac arrhythmias and spontaneous breathing efforts is a major limiting factor to their use in clinical practice. In critically ill spontaneously breathing human patients, the response to volume expansion can be predicted by ultrasound assessment of respiratory induced variations in inferior vena cava diameter (i.e., inferior vena cava collapsibility index) (36, 37). In a heterogeneous population of hospitalized dogs, ultrasonographic indexes showed different results, the “caudal” vena cava collapsibility index could not predict fluid responsiveness; while the “caudal” vena cava to aortic diameter ratio (CVC/Ao ratio), showed reasonable ability to discriminate responsiveness status *a priori* to volume expansion (19). The CVC/Ao ratio appears useful to detect early stages of volume depletion in dogs, but with variable results (38–41).

## The fluid Challenge Approach and the End-Point of Fluid Ressuscitation

A fluid challenge is the rapid administration of a relatively small volume of fluid to test if an individual has a cardiac preload reserve. Inasmuch as small amounts of fluids are titrated “to effect,” based on pre-established goals, the fluid challenge also provides volume replacement with lower risk of volume overload (9, 10). Percent changes in SV or CO are the target hemodynamic variables and individuals where SV or CO increased by more than 10–15% from values recorded before the fluid challenge are characterized as responders to volume expansion (i.e., are positioned in the ascending limb of the Frank-Starling Curve). With the fluid challenge traditionally used in adult humans (500 ml of artificial colloids or crystalloids), it has been recognized that 52% of patients in the ICU and 63% of patients in the operating room show preload dependency (i.e., are responders to volume expansion) (32). More recent a meta-analysis has confirmed that only 50% of critically ill human individuals positively respond to a fluid challenge (42).

In individuals with signs of poor tissue perfusion that are responders to the first fluid challenge (SV or CO increase ≥10–15% above baseline), maximization of SV and CO can be achieved by administering additional fluid challenges (9, 10). Before a decision is made on whether fluid resuscitation should be continued, SV or CO values recorded after the previous fluid challenge is taken as the new baseline to determine percent changes in SV or CO. Stepwise fluid resuscitation can be interrupted once the individual becomes a non-responder to volume expansion (i.e., until increases in SV or CO are <10–15%). Because the only excess fluid administered corresponds to the volume of one fluid challenge, the risk of volume overload can be minimized (9, 10, 42).

**Figure 2:**
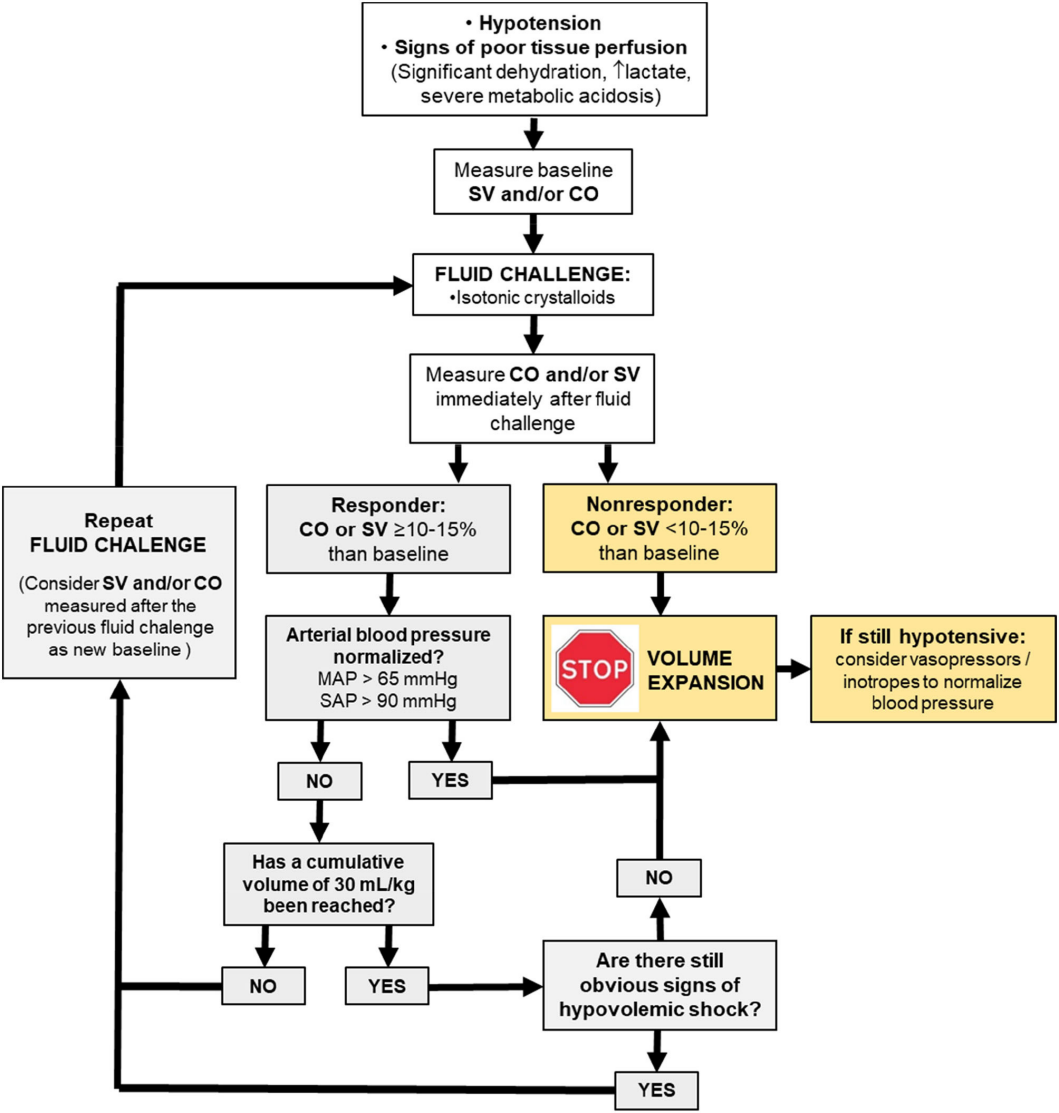
Physiologically driven fluid resuscitation protocol incorporating the fluid challenge approach to maximize stroke volume (SV) and cardiac output (CO). MAP, meant arterial pressure; SAP, systolic arterial pressure.

One question that remains opened is the end-point of fluid resuscitation in individuals that show preload dependence. Unless there is evidence of significant hypovolemia, it has been the suggested in humans admitted for emergency care that the volume of intravenous fluids should be initially limited to 20–30 ml/kg before vasopressors are considered to treat hypotension (7). The updated guidelines of the surviving sepsis campaign recommends intravenous administration of 30 ml/kg of crystalloids during the first hour in septic human patients with hypotension and elevated plasma lactate (43, 44). Although, fluids are titrated to maximize SV and CO in GDFT (i.e., until the individual is in the transition between the ascending and the flat portion of the Frank-Starling curve), a maximum cumulative volume of fluids can be predefined in GDFT protocols to minimize the risk of fluid overload (45). In patients that are hypotensive from hypovolemia and sepsis, normalization of arterial pressure could be determinant for terminating fluid resuscitation, even if assessment of fluid responsiveness still shows a state of preload dependence (45). Otherwise, vasopressor therapy should be initiated within the first hour if initial fluid administration is not sufficient to achieve the hemodynamic resuscitation goals (43–45) (Figure 2).

In individuals admitted for emergency care with signs of circulatory failure, hypotension is often caused by poor circulating volume that may be associated or not with sepsis-induced loss of vasomotor tone. Otherwise, hypotension during anesthesia is often caused by drug-induced decreases in systemic vascular resistance (SVR) and/or CO and does not imply that there is a circulating volume deficit (46). Although, it has been recognized that responders to volume expansion are not necessarily hypovolemic (28), hypotension-inducing hemorrhage in isoflurane anesthetized dogs can lead to a state of preload dependence, as suggested by increases in PPV and PVI from pre-hemorrhage values (47, 48). In case of intraoperative hypotension where the suspected cause is poor circulating volume, a fluid challenge approach may be attempted but cardiovascular support with sympathomimetics should not be delayed due to the dose-dependent depressant effects of inhalant anesthetics and/or neuraxial anesthesia on contractility and vasodilation (46). A drug with alpha-1 and beta-1 effects is ideal to improve both arterial blood pressure and CO, respectively (46). Constant rate infusions of vasopressor drugs, such as norepinephrine and dexmedetomidine, should be carefully used as these drugs can mask hypotension and increases in PPV and SPV caused by poor circulating volume, probably by shifting blood from the unstressed to stressed volume *via* a venoconstrictive action (47, 49).

According to a systematic review of literature, the fluid challenge most commonly used in adult human patients with signs of poor tissue perfusion consisted of 500 ml of crystalloids or colloids (~7 ml/kg to an adult patient weighing 70 kg), administered during 20–30 min (42). The target variable most commonly monitored after the fluid challenge in humans was an increase in CO ≥ 15% (42). Because traditionally used fluid challenges might result in some risk of volume overload, a mini-fluid challenge technique, based on the use of smaller volumes of fluids administered at faster rates has been used to predict if patients will respond to a “standard” fluid challenge (Table 1). Use of a mini-fluid challenge to test for a cardiac preload reserve can reduce the risk of fluid overload that would otherwise occur with the administration of a traditional fluid challenge. In critically ill adult humans, rapid administration of 50–150 ml of fluids during 10 s to 2 min reasonably predicts the response to larger volumes of fluids (250–500 ml) (50–55). In children undergoing elective surgery, however, a 3 ml/kg mini-fluid challenge administered during 2 min weakly predicted the effects of a 15 ml/kg volume of lactated Ringer's solution (LRS) (56). Recent meta-analysis concluded that a mini-fluid challenge reliably predicts fluid responsiveness status in the operating room and in the ICU setting (57).

**Table 1:** Clinical studies using a mini-fluid challenge to predict the response to a standard fluid challenge in humans.

**Mini fluid challenge:**				**Fluid challenge (criterion of responder)**	**Population evaluated**	**Reference**
**Fluid type**	**Volume (infusion time)**	Best cutoff^*^	AUROC^**^ (95% CI)			
6% HES	100 ml (1 min)	↑ VTI_aorticflow_ ≥ 10%	0.92 (0.78–0.98)	500 ml/15 min (↑ VTI_Aorticflow_ ≥ 15%)	39 patients with acute circulatory failure under mechanical ventilation.	Muller et al. (50)
“Crystalloid”	50 ml (10 s)	↑ VTI_Aorticflow_ > 9%	0.91 (NR)	500 ml/15 min (↑ CO_Echo_ ≥ 15%)	55 mechanically ventilated patients with inadequate perfusion.	Wu et al. (51)
“Crystalloid”	50 ml (10 s)	↑ CO_Echo_ > 6%	0.95 (NR)	500 ml/15 min (↑ CO_Echo_ ≥ 15%)	Same as above.	Wu et al. (51)
0.9% NaCl	100 ml (2 min)	↑ SVI_PCA_ > 6%	0.95 (0.90–0.99)	250 ml/10 min (↑ SVI_PCA_ ≥ 10%)	44 mechanically ventilated patients undergoing neurosurgery.	Biais et al. (52)
5% Albumin	150 ml (1 min)	↑ SV_Echo_ > 10%	0.86 (0.6–0.9)	500 ml/16 min (↑ SV_Echo_ ≥ 15%)	14 Child A cirrhosis patients receiving liver transplant^***^.	Mukhtar et al. (53)
0.9% NaCl	100 ml (1 min)	↑ SVI_PCA_ > 5.8%	0.97 (0.84–0.99)	500 ml/11 min (↑ SVI_PCA**_> 15%)	33 mechanically ventilated obese patients undergoing neurosurgery.	Ali et al. (54)
6% HES	100 ml (1 min)	↑ SV_PCA_ > 5%	0.90 (0.82–0.99)	500 ml/11 min (↑ SV_PCA**_≥ 15%)	50 patients undergoing spine surgery in prone position.	Lee et al. (55)
LRS	3 ml/kg (2 min)	↑ VTI_Aorticflow_ > 1.5%	0.77 (0.63–0.87)	15 ml/kg (10 min)	55 children under general anesthesia for elective surgery with high risk of bleeding.	Zorio et al. (56)

* *Best cutoff threshold to predict responders to the conventional fluid challenge*.

** *AUROC: Area under the receiver operating characteristics curve (values ≥ 0.75 and ≥ 0.90 indicate good and excellent ability to discriminate responders from non-responders to volume expansion, respectively)*.

*** *For individuals with child B and C cirrhosis the mini fluid challenge did not have discriminative ability (AUROC not different from 0.5)*.

*CI, confidence intervals; HES, hydroxyethyl starch; ↑, increase in variable; LRS, lactated Ringer's solution; VTI_Aorticflow_, velocity-time integral of aortic flow measured by echocardiography; CO_Echo_, cardiac output measured by echocardiography; SVI_PCA_, stroke volume index measured by uncalibrated pulse contour analysis; SV_Echo_, stroke volume measured by echocardiography; SV_PCA_, stroke volume measured by uncalibrated pulse contour analysis*.

A summary of studies evaluating responsiveness using fluid challenges in small animals is presented in Table 2. Several reports in veterinary medicine have evaluated fluid responsiveness *via* a fluid challenge approach in healthy/normovolemic dogs under anesthesia (12, 15, 16, 20–22). In other studies a fluid challenge has been administered in dogs anesthetized for orthopedic/oncologic surgery that presented intraoperative hypotension (mean arterial pressure <65 mmHg) (13, 18), or in dogs anesthetized for abdominal surgeries potentially associated with hemodynamic instability that were predicted as responders to volume expansion based on PPV values >13% (14).

**Table 2:** Fluid challenge techniques with crystalloids used to evaluate fluid responsiveness in dogs.

**Type of fluid**	**Volume infused (duration of infusion)**	**Criterion used to define responders to the fluid challenge**	**Percentage of responders to the first fluid challenge**	**Population evaluated**	**Reference**
LRS	5 ml/kg (1 min)	↑ VTI_Aorticflow_ ≥ 15%	38%	24 healthy dogs undergoing elective surgeries.	Bucci et al. (12)
LRS	15 ml/kg (15 min)	↑ CO_Echo_ ≥ 15%	76%	33 dogs with hypotension during anesthesia under mechanical ventilation for orthopedic surgery.	Fantoni et al. (13)
LRS	10 ml/kg (10–15 min)	↑ SV_EDM_ ≥ 10%	82%	35 dogs anesthetized under mechanical ventilation for abdominal surgeries with pulse pressure variation > 13%.	Drozdzynska et al. (14)
LRS	20 ml/kg (15 min)	↑ SVI_TPTD_ > 15%	100%	39 healthy bitches anesthetized under mechanical ventilation undergoing ovariohysterectomy.	Celeita-Rodríguez et al. (16)
LRS	4 ml/kg over (1 min)	↑ VTI_Aorticflow_ ≥15%	50%	26 conscious dogs referred for various clinical conditions.	Oricco et al. (17)
LRS	15 ml/kg (15 min)	↑ SV_Echo_ ≥ 15%	70%	50 dogs anesthetized under mechanical ventilation for orthopedic or oncologic surgery.	Gonçalves et al. (18)
LRS	4 ml/kg over (1 min)	↑ VTI_Aorticflow_ ≥ 15%	46%	22 dogs hospitalized for a variety of medical conditions.	Rabozzi et al. (19)
LRS	5 ml/kg (5 min)	↑ CI_PCA_ ≥ 15%	62.5%	80 dogs anesthetized under mechanical ventilation for orthopedic or soft tissue surgery.	Skouropoulou et al. (21)
LRS	10 ml/kg (5 min)	↑ SV_TPTD_ > 15%	83%	48 healthy dogs anesthetized under mechanical ventilation for elective surgery.	de Oliveira et al. (20)

*LRS, lactated Ringer's solution; ↑, increase in variable; VTI_Aorticflow_, velocity-time integral of aortic flow measured by echocardiography; CO_Echo_, cardiac output measured by echocardiography; SV_EDM_, stroke volume measured by esophageal Doppler monitor; SVI_TPTD_, stroke volume index measured by transpulmonary thermodilution; SV_Echo_, stroke volume measured by echocardiography; CI_PCA_, cardiac index measured by uncalibrated pulse contour analysis; SV_TPTD_, stroke volume measured by transpulmonary thermodilution*.

The volume and duration of infusion of fluid challenges administered to small animal patients in the literature varies considerably. Fluid responsiveness status in dogs has been assessed *via* administration of LRS at doses ranging from 10 to 20 ml/kg administered over 5–15 min (13, 14, 16, 18, 20, 22). In one study, a 10 ml/kg bolus of 6% hydroxyethyl starch (HES) was administered during 13 min in anesthetized dogs (15). Smaller volumes of isotonic crystalloids (LRS), administered at faster (4–5 ml/kg during 1 min) (12, 17, 19) or slower rates (5 ml/kg during 5 min) (21) have been used to evaluate fluid responsiveness status in canine species. Although, one might define these smaller volumes of LRS as mini fluid challenges, such definition might not be appropriate because there have been no published studies evaluating the ability of these volumes to predict the response to fluid challenges traditionally used in canine species (15–20 ml/kg of LRS).

The criteria used to define responders to volume expansion in veterinary studies has been an increase in CO or SV ≥10–15% (Table 2). Transthoracic echocardiography (12, 15, 17, 19), transesophageal echocardiography (13, 18), esophageal Doppler (14), transpulmonary thermodilution (16, 20, 22), and uncalibrated pulse contour analysis (21) have been used to monitor fluid responsiveness status in anesthetized dogs. While it has been recognized that only 50% of critically ill human individuals positively respond to a fluid challenge with substantial increases in CO or SV (32, 42), the percentage of responders to a single fluid challenge with crystalloids in canine studies has been reported to range from 38 to 100% (Table 2). This large variability could be partly related to the fact that fluid responsiveness was evaluated in animals presenting with varying clinical conditions, which may lead to an altered fluid responsiveness status due to varying degrees of systemic illness. The proportion of responders/non-responders to volume expansion in these studies could also have been influenced by the use of fluid challenges administered at different volumes and rates, and to the use of monitoring devices that differ in their ability accurately to detect CO and/or SV changes induced by volume expansion (58).

## Which Target Variable Should Be Used to Assess Fluid Responsiveness Status by a Fluid Challenge?

In spite of recommendations for monitoring CO or SV changes as the main response variable during GDFT, large scale clinical trials reported that arterial blood pressure was still the most commonly used target variable in humans (59). However, evaluation of changes in arterial pressure as a surrogate of CO changes could be misleading even in individuals presenting with hypotension from hypovolemia because arterial elastance/resistance can be significantly altered (20, 59, 60). Data in veterinary medicine is lacking, but it appears that arterial blood pressure is commonly used as the main target variable in dogs and cats admitted with signs of shock. Despite its limitations, arterial pressure is the preferred method to assess the response to a fluid challenge if measurement of SV and CO or its surrogates are not available. In veterinary medicine, systolic arterial pressure (SAP) measured by a Doppler ultrasound device has the advantage of allowing a subjective assessment of the quality of the peripheral perfusion. The pulsatile arterial blood flow detected by the Doppler ultrasound may be inaudible or muffled in dogs and cats with severe circulatory failure, but it often improves after a first fluid challenge, allowing measurement of SAP. In the presence of signs of shock (SAP <90 mmHg), an increase of at least 5–10 mmHg in SAP induced by a fluid challenge can be used to define a responder volume expansion. In a similar way as for SV and CO, as long as the response remains positive, fluid challenges are repeated until the target SAP is achieved. If an animal does not respond to a fluid challenge with increases in SAP, fluid resuscitation should be stopped and other alternatives to stabilize blood pressure considered. In a retrospective study of dogs admitted to a veterinary care facility with signs of circulatory failure (SAP <90 mmHg), when bolus fluid administration resulted in normalization of SAP within the first hour of fluid resuscitation (Doppler SAP ≥ 90 mmHg) there was lower probability of death compared to animals that remained hypotensive in response to rapid volume resuscitation (61).

The lack of implementation of CO or SV changes to guide fluid administration in patients with circulatory failure could be attributed to difficulties in monitoring CO and SV in a practical and reliable way. A clinically useful method of CO and SV measurement should be accurate (i.e., provide values that are close to a reference value), precise (i.e., provide values in close proximity when repeated measurements are performed during steady state conditions), and provide good ability to track changes in the target variable (62, 63). According to current standards, a new technique of CO measurement is interchangeable with a reference method if the alternative technique presents acceptable agreement and good trending ability with the reference standard (62, 63). Percentage error (PE), calculated as 2 times the standard deviation of the bias between methods divided by the average CO of the population, evaluates the precision of agreement between methods. It has been suggested that PE values <30% denote an acceptable agreement between the alternative method and the reference standard used to measure CO (62). Polar plot analysis evaluates ability of an alternative method to track changes in the reference standard based on directional changes (increase or decrease in CO) and on the magnitude of changes in CO, with mean polar angle ≤ ±5° and radial limits of agreement ≤ ±30° denoting good trending ability (63).

In addition to meeting the criteria of accuracy and precision, a device should also be operator independent and allow real time CO and SV monitoring *via* minimally invasive or non-invasive procedures (64). The accuracy/precision, trending ability, degree of invasiveness, operator dependence, and ability to provide real time CO and SV estimations of hemodynamic monitors currently available, with emphasis on their clinical application in fluid therapy guided by hemodynamic goals is discussed below.

### Indicator Dilution Cardiac Output Techniques

Indicator dilution techniques are relatively independent of the operator skill and are considered the most accurate and precise methods of CO measurement available for clinical use (65). However, due to varying degrees of invasiveness and some other technical difficulties, indicator dilution techniques have not been routinely implemented to evaluate fluid responsiveness during fluid resuscitation of patients with signs of circulatory failure. Indicator dilution techniques provide intermittent CO and SV monitoring that need to be updated by the generation of new indicator dilution curves *via* injection of a thermal or a chemical indicator, which could make evaluation of fluid responsiveness less practical.

#### Pulmonary Artery Thermodilution

Among the indicator dilution methods available, the pulmonary artery thermodilution (PATD) technique has been the clinical gold standard method for CO monitoring since its introduction in the 1970's (65). Pulmonary artery thermodilution measures right ventricular blood flow after a known volume of thermal indicator (ice-cold or room temperature 5% dextrose) is injected into the vena cava/right atrium for recording the change in blood temperature over time (thermodilution curve) in the pulmonary artery, *via* a fast-response thermistor located in the tip of a pulmonary artery catheter (PAC) (65). Cardiac output measured by PATD (CO_PATD_) was used to evaluate the response to a fluid challenge in mechanically ventilated human individuals with acute circulatory failure related to sepsis (23, 26, 30) or during anesthesia for major surgery (66). However, questionable improvement in patient outcome in face of the higher degree of invasiveness and the inherent risks involved with placement of a PAC, including intra-cardiac knotting of the catheter and pulmonary artery rupture/thrombosis, has fueled a debate on whether the PAC should still be used (67, 68). For these reasons, advanced hemodynamic monitoring by means of a PAC in human ICUs has declined in favor of other less invasive/non-invasive methods of CO monitoring (67–69). In veterinary medicine, insertion of PACs for hemodynamic monitoring has been largely restricted to experimental studies.

#### Transpulmonary Thermodilution

Introduced during the 1990's in the medical field, transpulmonary thermodilution (TPTD) is a less invasive indicator dilution technique than PATD because changes in blood temperature induced by the thermal indicator are measured by a thermistor tipped catheter inserted into a central artery of the systemic circulation (e.g., femoral artery) (65, 69). Experimental studies have shown that the TPTD technique shows good ability to track changes in CO. A strong correlation (correlation coefficient = 0.95) between TPTD and the gold standard reference method (perivascular flow probe around the pulmonary artery) has been reported during CO changes induced by hemorrhage and volume replacement (70).

A summary of studies in small animals evaluating the ability of CO measured by TPTD (CO_TPTD_) to track changes in CO measured by the reference standard (CO_PATD_) using polar plot analysis is presented in Table 3 (71–73). Based on experimental studies in dogs, where a range of CO values was obtained by increasing depth of anesthesia and inotropic (dobutamine) administration, it has been suggested that CO_TPTD_ can be used in replacement of CO_PATD_ because of an acceptable precision of agreement and good trending ability between these two techniques (73). Another report showed a marginal trending ability between CO_TPTD_ and CO_PATD_ in healthy anesthetized dogs (72). However, results were limited by the fact that the anesthetic protocol was designed for another purpose and no specific procedures were made to induce changes in CO (72). In cats, limited data from a small number of animals (n = 3) showed that CO_TPTD_ overestimated CO_PATD._ However, there was an acceptable precision of agreement and trending ability based on polar plot analysis (71).

**Table 3:** Ability of several cardiac output monitors to track changes in cardiac output measured by a reference method induced by in dogs and cats.

**Test method**	**Reference method**	Concordance rate^*^	Mean polar angle (radial LOA)^**^	**Trending ability**	**Species (number of animals) procedures/interventions**	**Reference**
CO_TPTD_	CO_PATD_	94%	−5° (±33°)	Acceptable	Cats (*n* = 3) Inotropes, vasopressors, increased depth of anesthesia to manipulate CO	Kutter et al. (71)
CO_TPTD_	CO_PATD_	94%	−12° (±35°)	Marginal	Dogs (*n* = 6) Anesthesia for pharmacokinetic study (no procedures aiming to change CO).	Kutter et al. (72)
CO_TPTD_	CO_PATD_	100%	2° (±12°)	Good	Dogs (*n* = 8) Inotropes/increased depth of anesthesia to manipulate CO (Thermal signal: 5 ml of ice-cold 0.9% NaCl).	Garofalo et al. (73)
CO_TPTD_	CO_PATD_	100%	−1° (±8°)	Good	Dogs (*n* = 8) Inotropes/increased depth of anesthesia to manipulate CO (Thermal signal: 10 ml of ice-cold 0.9% NaCl).	Garofalo et al. (73)
Calibrated CO_PCA_ (PICCO^2^)	CO_PATD_	82%	−10° (±46°)	Poor	Cats (*n* = 3) Inotropes, vasopressors, increased depth of anesthesia to manipulate CO.	Kutter et al. (71)
Calibrated CO_PCA_ (PICCO^2^)	CO_PATD_	77%	−11° (±57°)	Poor	Dogs (*n* = 6) Anesthesia for pharmacokinetic study (no procedures aiming to change CO).	Kutter et al. (72)
Calibrated CO_PCA_ (PICCO^2^)	CO_PATD_	63%	38° (±33°)	Poor	Dogs (*n* = 8) Vasodilation (nitroprusside) and vasoconstriction (phenylephrine).	Garofalo et al. (73)
Calibrated CO_PCA_ (PulseCO)	CO_PATD_	74%	2° (±60°)	Poor	Dogs (*n* = 6) Anesthesia for pharmacokinetic study (no procedures aiming to change CO).	Kutter et al. (72)
Uncalibrated CO_PCA_ (PRAM)	CO_PATD_	93%	3.9° (±12.1°)	Good	Dogs (*n* = 6) Anesthesia for abdominal surgery. (Dogs with arrhythmias or that received sympathomimetics excluded).	Briganti et al. (74)
Electrical velocimetry	CO_PATD_	88%	−5.9° (±46°)	Poor	Beagle dogs (*n* = 7) Anesthesia with sevoflurane under mechanical ventilation for experimental open-chest cardiovascular surgery.	Sasaki et al. (75)
Pulsed wave transit time	SV_PATD_	95%	NR	Acceptable	Dogs (*n* = 8) Anesthesia with isoflurane under mechanical ventilation. Vasopressors, inotropes, and increased depth of anesthesia to manipulate CO.	Sano and Chambers (76)

* *Concordance rates based on four-quadrant plot analysis: >95, between 90 and 95%, and <90%, indicate good, acceptable (or marginal), and poor trending ability, respectively (63)*.

** *Mean polar angles and radial limits of agreement based on polar plot analysis: mean polar angle ≤ 5° and radial limits of agreement ≤ 30° indicate good trending ability (63)*.

*CO_TPTD_, cardiac output measured by transpulmonary thermodilution; CO_PATD_, cardiac output measured by pulmonary artery thermodilution; CO_PCA_, cardiac output measured by pulse contour analysis, SV_PATD_, stroke volume measured by pulmonary artery thermodilution; NR, not reported*.

Investigations have used the TPTD technique as a reference method to determine “true” fluid responsiveness status in humans (58, 77–79) and, more recently, in veterinary medicine (16, 20, 22). Averaging triplicate CO_TPTD_ measurements results in adequate precision (i.e., variability of repeated measurements <10%) to evaluate fluid responsiveness. The minimum percent change in CO that can be trusted as significant and not related to the imprecision of the method [i.e. the least significant change (LSC)] was reported decrease from 20% with single CO_TPTD_ measurements to 12% with triplicate CO_TPTD_ measurements in humans, which is adequate to detect responders to volume expansion (CO increases ≥ 15%) (80). Averaging the three closest sequential CO_TPTD_ values from a series of five measurements in anesthetized dogs, resulted in a higher level of precision (LSC = 5.1%) than previously reported in humans (LSC = 12%), which could allow reliable detection of changes in CO ≥ 10% (16).

In spite of its acceptable accuracy/precision, CO_TPTD_ monitoring during the fluid resuscitation phase is limited by the fact that it requires not only a central venous catheter for injection of the thermal indicator (ideally ice-cold physiological saline), but also a thermistor tipped catheter placed in a central artery (e.g., femoral artery) (65, 69). Although, placing the thermistor tipped catheter in a peripheral artery seems an attractive alternative, this procedure is not recommended because loss of thermal signal in the periphery will lead to errors in CO measurement (overestimation of CO due to a decrease in the area under the thermodilution curve). Placement of the thermistor tipped catheter in a peripheral (metatarsal) artery in dogs failed to generate thermodilution curves necessary to measure CO_TPTD_ (81). Another disadvantage is the fact that CO_TPTD_ monitoring does not provide real time CO assessment and the need for rapid injection of ice-cold thermal signal to generate 3 thermodilution curves can result in an additional intravascular volume load. For use in small animals, the TPTD technique could be further limited by the high cost of the thermodilution catheter, which was designed for single use. Also the risk of displacement of the catheter from its insertion site (femoral artery) and hematoma formation should be considered as potential complications (72).

#### Lithium Dilution

Lithium dilution is a less invasive technique compared to PATD and TPTD because it does not demand catheterization of a central vein or a central artery. Injection of the chemical indicator (lithium chloride) can be performed through a peripheral venous catheter in humans and dogs (82, 83). The lithium dilution curve (plot of the lithium concentration over time) is measured in arterial blood withdrawn from a peripheral artery catheter into a lithium sensor located outside the patient (84). Although, PE (precision of agreement) calculations have not been reported in earlier studies, subjective assessment of Bland Altman analysis results has led authors to conclude that this technique provides reasonably good agreement with CO_PATD_ in humans and animals, including dogs and cats (82–87). However, in addition to the fact that this technique demands catheterization of a peripheral artery (which may carry greater difficulty in hypovolemic/hypotensive patients), repeated lithium chloride injections for measuring CO in hemodynamically unstable patients may lead to inaccurate results due to lithium accumulation/recirculation (86). The lithium dilution technique does not seem a suitable technique for repeatedly assessing fluid responsiveness status in smaller veterinary patients, such as cats and small dogs, because the amount of arterial blood withdrawn into the lithium sensor (~4.6 ml per measurement) can become significant if several measurements are performed over time (87).

### Pulse Contour Analysis Cardiac Output Techniques

While indicator dilution techniques provide intermittent CO values that need to be updated by the generation of new indicator dilution curves *via* injection of additional amounts of thermal (e.g., ice-cold 5% dextrose) or chemical (lithium chloride) indicators, the pulse contour analysis (PCA) method provides continuous, real time estimation of CO and SV.

Real time estimation of CO and SV could be particularly convenient to evaluate the response to a fluid challenge during volume resuscitation (88). However, PCA methods with potential application in animals demand catheterization of a central or a peripheral artery, which may carry greater difficulty in animals with signs of shock. The major limitation of PCA devices is the poor ability to track changes in CO during conditions of hemodynamic instability associated with changes in SVR in humans (89, 90) and animals (72, 73, 91). Studies evaluating the ability of calibrated and uncalibrated PCA monitors to track changes in CO_PATD_ in small animals are summarized in Table 3. Interpretation of these studies should consider if their design incorporated or not manipulations aiming to induce changes in SVR, since the performance of PCA methods can be altered by hemodynamic instability associated with vasoconstriction/vasodilation.

#### Calibrated Contour Analysis Methods

The same monitors that measure CO intermittently by indicator dilution techniques (TPTD and lithium dilution) also provide real time CO and SV estimations by the calibrated PCA method. This technique is based on analysis of the arterial pressure waveform to estimate real time CO and SV using proprietary algorithms, after calibration of the system with CO_TPTD_ (PiCCO^2^ system and VolumeView/EV1000 system) or with CO measured by lithium dilution (LIDCOplus and PulseCO systems). Studies have suggested that the calibrated PCA method could be useful to evaluate the response to a fluid challenge in humans (77, 79, 92). Cardiac output measured by a calibrated PCA system (PiCCO^2^) showed good correlation with changes in CO_PATD_ and CO_TPTD_ induced by volume expansion (correlation coefficient ≥ 0.70) (77, 92).

The ability of less invasive and non-invasive methods of CO monitoring to discriminate fluid responsiveness status is presented in Table 4. In septic human patients, receiver operating characteristics (ROC) curve analysis showed that continuous CO measured by calibrated PCA (PICCO^2^ system) could discriminate responders (CO_TPTD_ increases ≥ 15%) from non-responders (CO_TPTD_ increases <15%) to volume expansion (77, 79). However, CO and SV estimations by calibrated PCA devices may become unreliable in case of significant changes in SVR (73, 89) and the ability of calibrated PCA devices to track changes in CO_TPTD_ induced by a fluid challenge has been reported as poor (58). Although, the drift in CO estimations can be corrected by frequent recalibration of calibrated PCA systems with the indicator dilution method (94), these devices may not meet minimum criteria of accuracy, precision, and trending ability to detect changes in CO_TPTD_ induced by a fluid challenge (58).

**Table 4:** Ability of less invasive/non-invasive methods of cardiac output and stroke volume monitoring to discriminate responders from non-responders to volume expansion induced by a fluid challenge.

**Less invasive/non-invasive monitor**	**Type of fluid (volume/infusion time)**	Best cutoff^*^	AUROC^**^ (95% CI)	**Reference method (% change that defined responders)**	**Population evaluated**	**Reference**
Calibrated CO_PCA_ (PiCCO^2^)	0.9% NaCl (500 ml/30 min)	≥12%	0.878 (0.736–0.960)	Transpulmonary thermodilution (↑ CO_TPTD_ ≥ 15%)	80 human patients with septic circulatory failure.	Monnet et al. (77)
Calibrated CO_PCA_ (PiCCO^2^)	6% HES (500 ml/15 min)	≥9%	0.85 (0.76–0.92)	Transpulmonary thermodilution (↑ CO_TPTD_ ≥ 15%)	78 human patients (after elective cardiac surgery).	Fischer et al. (79)
Non-calibrated CO_PCA_ (FloTrac/Vigileo)	NaCl 0.9% (500 ml/30 min)	≥12%	0.564^***^ (0.398–0.720)	Transpulmonary thermodilution (↑ CO_TPTD_ ≥ 15%)	80 human patients with septic circulatory failure.	Monnet et al. (77)
Non-calibrated CO_PCA_ (FloTrac/Vigileo)	NaCl 0.9% (500 ml/30 min)	NR	^***^	Transpulmonary thermodilution (↑ CO_TPTD_ ≥ 12%)	20 human patients with circulatory failure.	Monnet et al. (78)
Pulsed wave transit time	Crystalloid solution (500 ml/20 min)	≥11%	0.84 (0.69–0.99)	Transthoracic echocardiography (↑ CO_Echo_ ≥ 15%)	25 human patients in the early phase of septic shock.	Feissel et al. (93)
VTI_aorticflow_	LRS (10 ml/kg/5 min)	≥14.7%	0.901 (0.812–0.990)	Transpulmonary thermodilution (↑ SV_TPTD_ > 15%)	48 healthy dogs anesthetized for elective surgery.	de Oliveira et al. (20)

* *Best cutoff threshold to discriminate responders to the fluid challenge*.

** *AUROC: Area under the receiver operating characteristics curve (values ≥0.75 and ≥0.90 indicate good and excellent ability to discriminate responders from non-responders to volume expansion, respectively)*.

*** *AUROC not different from 0.5 (no discriminatory ability)*.

*CI, confidence intervals; NR, not reported; ↑, increase in reference method; CO_PCA_, cardiac output measured by pulse contour analysis; CO_TPTD_, cardiac output measured by transpulmonary thermodilution; CO_Echo_, cardiac output measured by echocardiography; VTI_Aorticflow_, velocity-time integral of aortic flow measured by echocardiography; SV_TPTD_, stroke volume measured by transpulmonary thermodilution*.

#### Uncalibrated Pulse Contour Analysis Methods

Real time CO estimations can be provided by analysis of the arterial pressure waveform without requiring previous calibration of the system with a reference technique [FloTrac/Vigileo, LiDCOrapid, pulse recording analytical method (PRAM), ProAQT/Pulsioflex]. Real time SV monitoring with an uncalibrated PCA device (ProAQT/Pulsioflex) was used to evaluate the ability of a mini fluid challenge (50 and 100 ml of physiological saline) to predict the response to 250 ml of physiological saline in mechanically ventilated human patients undergoing neurosurgery (52). However, uncalibrated PCA devices may not discriminate fluid responsiveness status based on ROC curve analysis (77, 78) (Table 4), and have shown poor ability to track changes in CO induced by a fluid challenge (58, 78, 95, 96) (Table 5).

**Table 5:** Ability of several monitors to track changes in cardiac output induced by a fluid challenge in humans.

**Test method**	**Reference method**	**Type of fluid (volume/duration of infusion)**	Concordance rate^*^	Mean polar angle (radial LOA)^**^	**Trending ability**	**Reference**
Uncalibrated CO_PCA_ (PRAM)	CO_Echo_	0.9% NaCl (500 ml/15 min)	60%	NR	Poor	Biais et al. (95)
Uncalibrated CO_PCA_ (FloTrac/Vigileo)	CO_TPTD_	0.9% NaCl (500 ml/15 min)	73%	NR	Poor	Monnet et al. (78)
Uncalibrated CO_PCA_ (FloTrac/Vigileo)	CO_TPTD_	6% HES (500 ml/20 min)	80.5%	7.4° (±43.5°)	Poor	Geisen et al. (58)
Uncalibrated CO_PCA_ (LiDCOrapid)	CO_TPTD_	Same as before	79.2%	10.5 (±41.7°)	Poor	Geisen et al. (58)
Calibrated CO_PCA_ (PiCCO^2^)	CO_TPTD_	Same as before	78.3%	−6.4° (±41.2°)	Poor	Geisen et al. (58)
Uncalibrated CO_PCA_ (PRAM)	CO_EDM_	“Crystalloid” (250–500 ml <10 min)	74%	7.8° (±41.7°)	Poor	Barthélémy et al. (96)

* *Concordance rates based on 4-quadrant plot analysis: >95, between 90 and 95%, and <90%, indicate good, acceptable (or marginal), and poor trending ability, respectively (63)*.

** *Mean polar angles and radial limits of agreement based on polar plot analysis: mean polar angle ≤ 5° and radial limits of agreement ≤ 30° indicate good trending ability (63)*.

*LOA, limits of agreement; NR, not reported; CO_PCA_, cardiac output measured by pulse contour analysis; CO_Echo_, cardiac output measured by echocardiography; CO_TPTD_, cardiac output measured by transpulmonary thermodilution; CO_EDM_, cardiac output measured by esophageal Doppler monitor*.

In spite of continuous changes in the algorithm of one uncalibrated PCA device (FloTrac/Vigileo) over the recent years, studies have shown a poor agreement and limited ability to track phenylephrine-induced changes in CO measured by echocardiography (97) and by PATD (98). Studies point out that this uncalibrated PCA device overestimates CO measured by indicator dilution methods (PATD and Lithium dilution) and is unreliable to monitor CO in dogs (99, 100).

For another uncalibrated PCA device (PRAM), there are conflicting results regarding its ability to track changes in CO measured by a reference method. Earlier studies in pigs, where a range of CO values were induced by hemorrhage and inotropic administration (dobutamine), reported an acceptable agreement (PE <30%) between PRAM and CO measured by an aortic flow probe and between PRAM and PATD (101). However, this uncalibrated PCA device has also shown an unacceptable agreement/poor ability to track changes in CO measured by thermodilution techniques in humans and in animals (90, 92). Other studies have reported that this device was unable to track changes in CO induced by volume expansion in humans (95, 96). In veterinary medicine, a preliminary study performed in anesthetized dogs reported that the PRAM method showed good precision, acceptable concordance, and good trending ability with CO_PATD_ (74). However, results were limited by the fact that its reliability was not determined during conditions of hemodynamic instability/changes in SVR.

### Echocardiography

**Figure 3:**
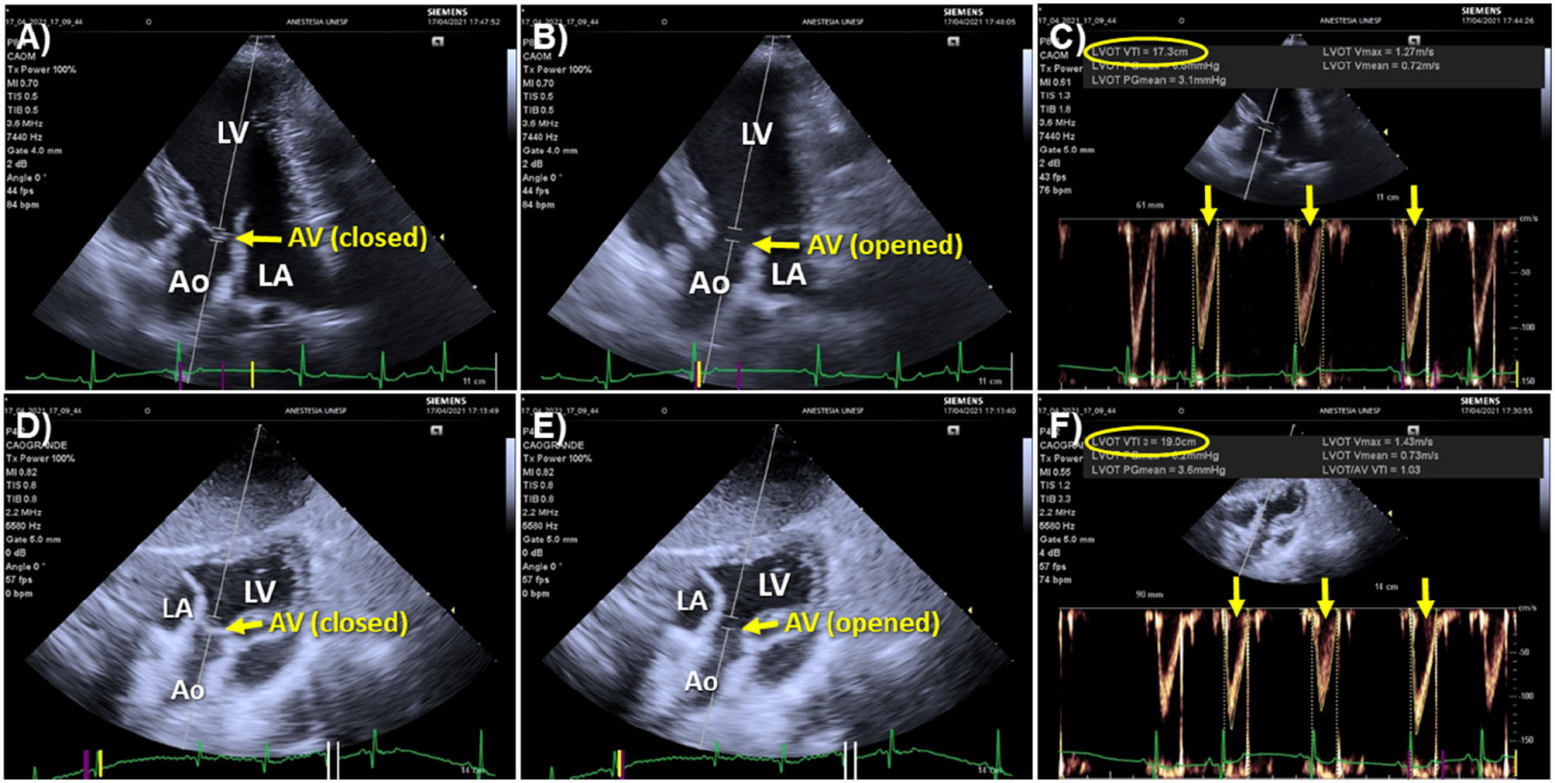
Echocardiographic windows used to measure aortic flow velocity-time integral (VTI) in dogs. **(A)** Left parasternal, apical five-chamber view optimized for visualizing the left ventricular outflow tract (LVOT) with the aortic valve closed during diastole. The Doppler beam is aligned as parallel as possible with the aortic outflow and the Doppler sampler is positioned just below the mitral annulus (point of insertion of aortic valve leaflets). **(B)** The same image with the aortic valve (AV) opened during systole. **(C)** Acceleration of aortic flow over time measured by positioning the Doppler cursor as shown in **(A,B)**. The aortic flow VTI averaged of 3 sequential heartbeats (yellow arrows) using an automated algorithm was 17.3 cm (yellow oval). **(D)** Subxiphoid view of the LVOT showing alignment of the Doppler beam with the aortic outflow and the placement of the Doppler sampler with the AV closed during diastole. **(E)** The same image with the AV opened during systole. **(F)** Measurement of aortic flow VTI according to the subxiphoid view. The VTI averaged from 3 sequential heartbeats (yellow arrows) was 19.0 cm (yellow oval). Comparison between the five-chamber **(A,B)** and subxiphoid views **(D,E)** shows that the latter allowed better alignment of the Doppler beam with the aortic outflow, which contributed to a higher VTI measured from the subxiphoid view. Ao, aorta; AV, aortic valve; LV, left ventricle; LA, left atrium.

Point of care echocardiography, in addition to its role in guiding fluid challenge administration, has the unique advantage of allowing to rule out other causes of shock, such as circulatory failure caused by pericardial effusion or by decreased myocardial contractility/systolic dysfunction, where fluid resuscitation is contraindicated. Cardiac output and SV measured by echocardiography (CO_Echo_ and SV_Echo_, respectively) can be used to evaluate the response to a fluid challenge. While transthoracic echocardiography is useful in conscious individuals with signs of circulatory failure, transesophageal echocardiography is a practical means of monitoring the response to a fluid challenge in anesthetized patients during surgery or in unconscious/sedated individuals under mechanical ventilation. According to this technique SV_Echo_ is firstly measured by multiplying the area of the left ventricular outflow tract (LVOT) during mid systole (the point of maximal opening of aortic valve leaflets) by the velocity time integral (VTI) of the LVOT. Because the LVOT area during mid systole does not to change over the respiratory and cardiac cycle, one single measurement of the LVOT area is usually performed for a patient as SV will vary exclusively as function of changes in aortic flow VTI (102–104). Parallel alignment between the Doppler beam and the aortic outflow, with an angle between the Doppler and LVOT as close to 0° as possible (ideally <20°) is critical for obtaining reliable aortic outflow measurements by echocardiography (105). The left parasternal five-chamber view has been used to evaluate aortic flow VTI in dogs and cats (106). However, in canine species, a subcostal (or subxiphoid) view may be preferable in point-of-care settings because it does not demand the use of a echocardiography examination table and more accurately measures peak aortic velocities, resulting in higher VTI values than the five-chamber view (106) (Figure 3).

Velocity time integral measurements are sensitive to operator experience, cardiac arrhythmias and heart valve defects. Given the fact that echocardiographic measurements are highly operator dependent, a question frequently raised is the need for a certified specialist to reliably monitor CO_Echo_. Studies have shown that CO_Echo_ measured by physicians with basic training in critical care echocardiography present good reproducibility and acceptable precision of agreement (PE = 17%) with CO_PATD_ (107). Providing that correct LVOT area and aortic flow VTI measurements are performed, transthoracic echocardiography has been considered an accurate and precise technique (PE <30%) to measure CO when compared with CO_PATD_ in critically ill humans (105). Polar plot analysis has also shown that CO measured by transthoracic echocardiography presents a good ability to track changes in CO_PATD_ (105), which is an important prerequisite for a technique used to assess the response to a fluid challenge. Beat by beat irregularities of left ventricular SV caused by arrhythmias will lead to a substantial error if CO_Echo_ and SV_Echo_ is calculated from one single VTI measurement. Accuracy of measurements can be improved by averaging as many VTIs as possible in the presence of heart rhythm irregularities (108). Cardiac output measured by trans-esophageal echocardiography has been shown to present a good precision of agreement with CO_PATD_ (PE <30%) in dogs that were normotensive and hypotensive during anesthesia (109). The ability of transthoracic and transesophageal echocardiography to track changes in CO_PATD_ has not been reported in small animals.

Cardiac output and SV values derived from transthoracic or transesophageal echocardiography have been used to discriminate responders from non-responders to volume expansion in the human medical field (25, 51, 53, 95, 110) and in dogs (13, 18). Because SV will change only as a function of changes in aortic flow, percent changes in aortic flow VTI have been used as a surrogate of SV to evaluate fluid responsiveness status in humans (36, 50, 56, 111) and dogs (12, 17, 19). More recently, in addition to the use of aortic flow VTI to track changes in SV, use of the “minute distance,” calculated as the VTI times the heart rate, has been proposed as a surrogate of CO in point-of-care settings (104).

Although, percent changes in aortic flow VTI have been used as a surrogate of SV changes in GDFT protocols, there have been no studies evaluating the ability of VTI to track changes in SV determined by a reference method (e.g., thermodilution CO). It must be borne in mind that the reliability of VTI measurements is highly operator dependent. To account for operator related errors, studies evaluating fluid responsiveness with echocardiography should ideally report intra and inter-operator agreement (112). The reliability of repeated measures performed by one single observer (repeatability) and between observers (reproducibility) can be assessed by intraclass correlation coefficients. Values between 0.6–0.74 and 0.75–1 suggest good and excellent correlation, respectively (112).

When VTI is used to evaluate fluid responsiveness, one important question that arises is the level of precision (i.e., the proximity of repeated measurements) of aortic flow measurements obtained during stable hemodynamic conditions. If the precision of the method is poor, it may not be able to correctly identify responders to volume expansion (80). In human patients with a regular heart rhythm, averaging the VTI from 3 sequential heartbeats is recommended for achieving an adequate precision (108). In the presence of a regular heart rhythm, the minimum percent change in VTI between two successive measurements that can be trusted as significant (i.e., the least significant change) was 11%, which would be adequate to detect increases in VTI ≥ 15% induced by a fluid challenge (108). Otherwise, in individuals with an irregular heart rhythm, evaluation of fluid responsiveness by VTI carries greater difficulty because of beat-by-beat variations on SV and aortic flow. Under these circumstances, averaging VTI from 5 sequential heartbeats or more may be necessary to achieve an adequate level of precision of the method (108).

The ability of a method to detect changes in SV induced by a fluid challenge can be inadequate during conditions of hemodynamic instability/irregular heart rhythm because the method's variance is composed not only by the precision of the method but also by the method's general variability about the true values (defined as “trueness”) (113). Therefore, relatively small physiologic variations in CO and SV values can worsen the precision of the method and impair the ability of VTI to detect smaller changes in CO and SV induced by a fluid challenge (113). Fear, anxiety and discomfort caused by physical restraining, which is often necessary performing transthoracic echocardiography in conscious animals, can result in changes in sympathetic tone and interfere with the level of precision of VTI measurements. In anesthetized dogs, percent changes in aortic flow VTI induced by a fluid challenge > 14.7% showed good ability to discriminate responders to volume expansion, defined as dogs where SV measured by TPTD increased > 15% after the fluid challenge (20) (Table 4). The close proximity between the optimal cutoff threshold determined from the ROC curve (percent change in VTI > 14.7%) and the percent change in SV used to define “true” responders to volume expansion, suggest that changes in aortic flow VTI can be used as a surrogate of SV to evaluate fluid responsiveness in dogs (20). However, percent changes in VTI associated with a high probability of false negative and/or false positive results (zone of diagnostic uncertainty) was reported to range from 11.8 to 17.6%, which could be related to errors in obtaining proper alignment of the pulsatile Doppler with the aortic flow (20). In conscious dogs with a variety of clinical conditions, baseline aortic flow VTI (measurements obtained prior to a fluid challenge) ≤ 10.3 cm predicted responders to volume expansion with high sensitivity (84.9%) and specificity (100%) (17).

**Figure 4:**
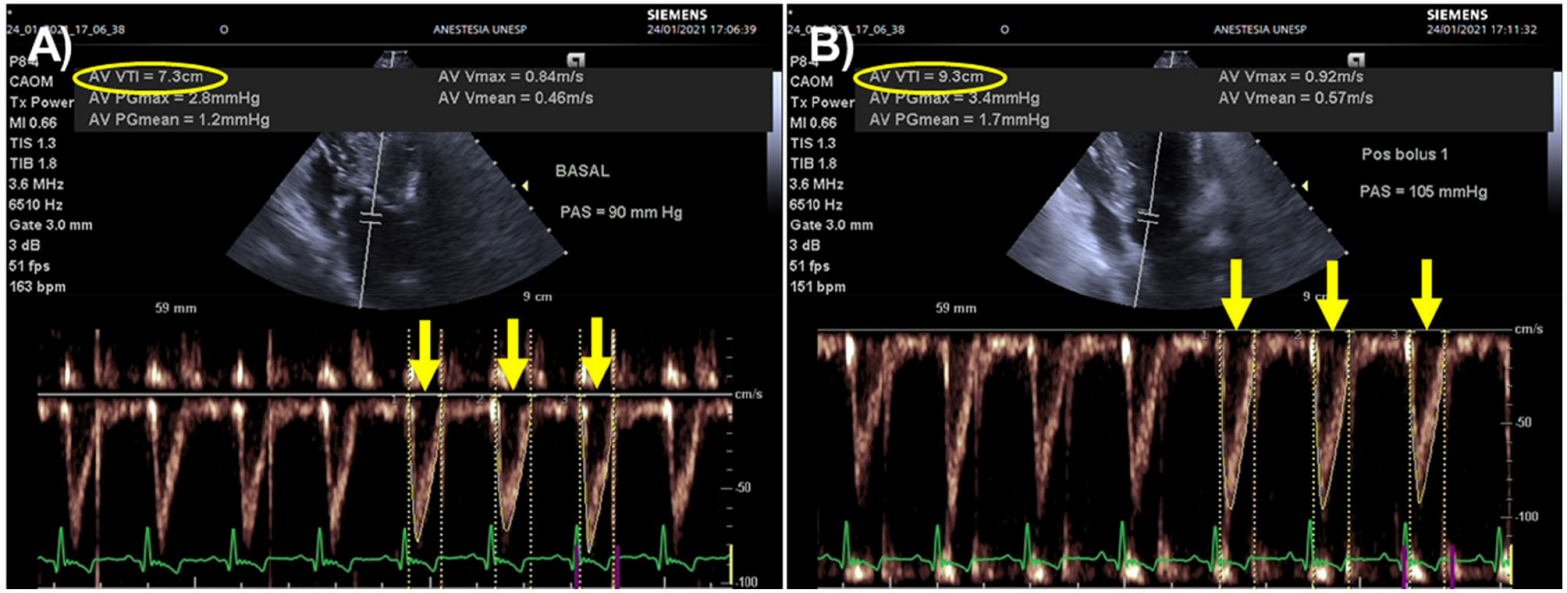
Effects of a fluid challenge with 10 ml/kg of lactated Ringer's solution administered over 5 min on aortic flow velocity time integral (VTI) recorded from an apical five-changer view in a dog with blunt trauma. An automated algorithm was used to measure aortic flow VTI as the average of 3 sequential heartbeats (yellow arrows). **(A)** Heart rate (HR), Doppler systolic arterial pressure (SAP), and aortic flow VTI before the fluid challenge were 163 beats/min, 90 mmHg and 7.3 cm (yellow oval), respectively. **(B)** After the fluid challenge, HR, Doppler SAP and aortic flow VTI were 151 beats/min, 105 mmHg and 9.3 cm (yellow oval). This animal was a responder to volume expansion because aortic flow VTI increased 27% (from 7.3 to 9.3 cm). The minute distance (HR times VTI), a surrogate index used to track changes in cardiac output, increased 17% after the fluid challenge (from 1197 to 1404 cm).

Measuring the VTI demands several keystrokes and could be time-consuming, which may be less practical in the emergency setting. However, with the recent development of algorithms incorporated into ultrasound machines, it has become possible to perform automated VTI measurements. Use of tools that allow automated VTI measurements has the potential for optimizing evaluation of fluid responsiveness in the emergency settings (Figure 4).

**Figure 5:**
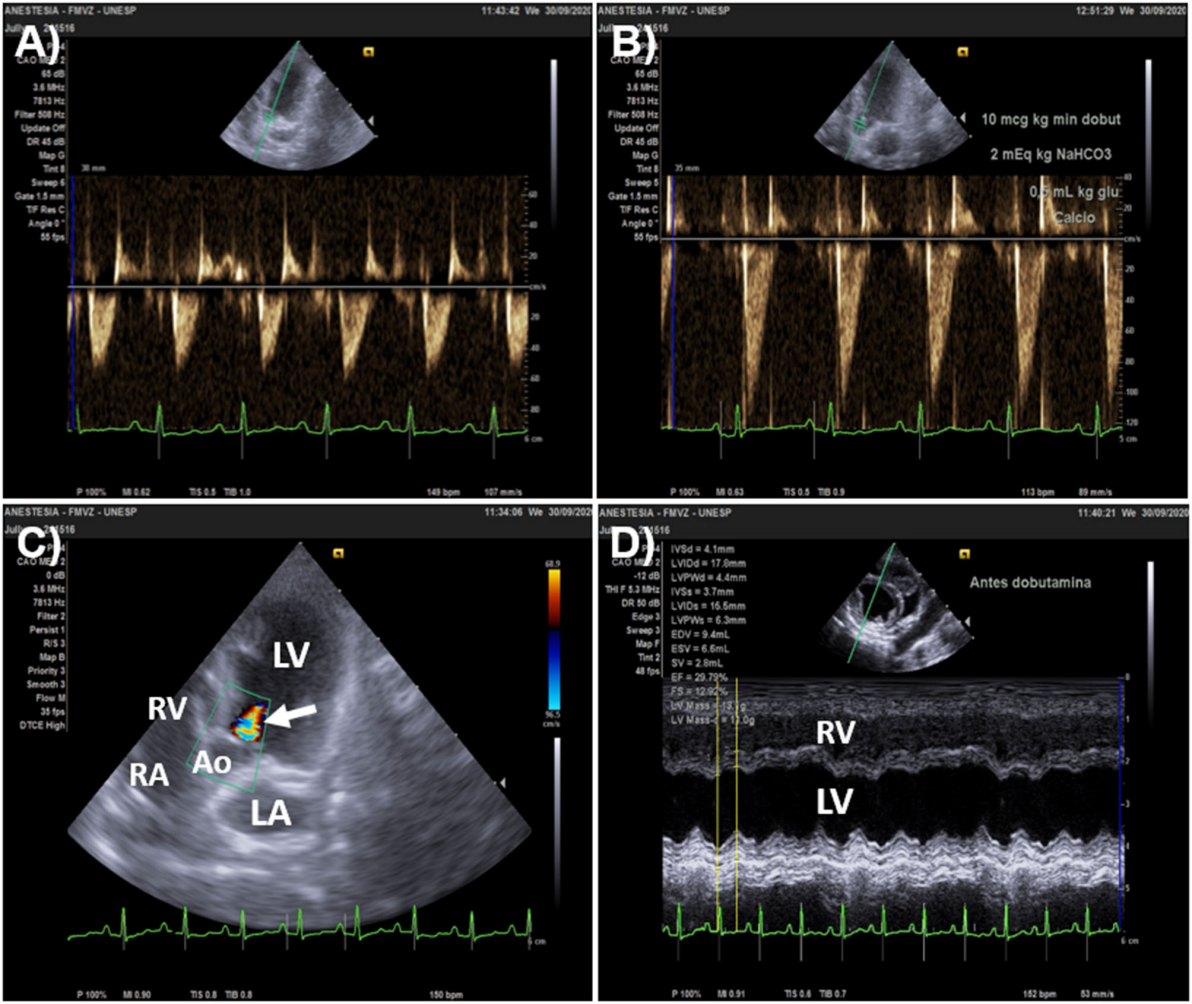
Overestimation of stroke volume (SV) changes by aortic flow velocity-time integral (VTI) in a 3.0 kg Pinscher dog that presented aortic regurgitation. The animal was admitted with signs of acute circulatory failure (unconsciousness of sudden onset, absent peripheral pulses and muffled heart sounds). **(A)** Baseline heart rate (HR) and aortic flow VTI (left parasternal, five-chamber apical view) was 149 beats/min and 5.1 cm, respectively. Doppler systolic arterial pressure (SAP) was undetectable. The minute distance (HR times VTI) was 760 cm. **(B)** After dobutamine was administered by constant rate infusion (10 μg/kg/min), HR was 113 beats/min and aortic flow VTI increased by 100% (from 5.1 to 10 cm); while minute distance and SAP were 1113 cm 170 mmHg, respectively. **(C)** The 100% increase in VTI detected after dobutamine overestimated percent increases in SV because of a significant aortic regurgitation shown by color Doppler (arrow). **(D)** M-mode echocardiography of the short axis of the heart (right parasternal view) upon admission showed that ejection fraction (EF) and fractional shortening (FS) were 29.8 and 12.9%, respectively, denoting severe systolic dysfunction (defined as EF <40% and FS <20%). Inotropic support was initially chosen to increase SV and cardiac output instead of fluid challenge administration in this animal because of severe left ventricular systolic dysfunction. Ao, aorta; RV, right ventricle; RA, right atrium; LA, left atrium; LV, left ventricle.

Velocity time integral measurements are overestimated in the presence of moderate-to-severe aortic regurgitation and sub-aortic obstruction (aortic stenosis) (104). Dynamic LVOT obstruction may be observed during severe hypovolemia/inotropic stimulation, in dogs with sub-aortic stenosis, and cats with hypertrophic cardiomyopathy. In the presence of these conditions, it is not possible to determine if an increase in aortic flow VTI results from increased SV or from increased regurgitant volume (aortic regurgitation) or from subaortic/LVOT stenosis (104) (Figure 5).

### Esophageal Doppler

The esophageal Doppler technique measures blood flow velocity in the descending aorta (or caudal aorta in quadrupeds) *via* a Doppler transducer inserted into the thoracic esophagus. The velocity of blood flow in the descending aorta plotted over time is used to determine the stroke distance (distance of the envelope that contains blood flow velocity vs. time curve measured in centimeters) (114–116). The SV measured by the esophageal Doppler method (SV_EDM_) is calculated by stroke distance times the cross sectional area of the aorta. A nomogram developed for human patients (based on body weight, height and body surface area) estimates the cross sectional area of the aorta, assuming a 45° angle between the aorta and the esophageal probe (114–116). The algorithm for SV determination also assumes that the proportion of the blood flow/SV generated by the left ventricle at each heartbeat to the descending aorta remains fixed at 70% and CO measured by the esophageal Doppler method (CO_EDM_) is calculated by the multiplying SV/stroke distance by the heart rate (114–116).

The esophageal Doppler monitor showed poor precision of agreement with the reference standard (PATD) in pediatric patients with congenital heart defects and in patients undergoing coronary artery bypass surgery (PE = 54 and 70%, respectively) (114, 115). Although, CO_EDM_ cannot be considered interchangeable with CO_PATD_, this device can show an acceptable/marginal trending ability with CO_PATD_, suggesting that this method could be used to guide volume expansion in goal directed fluid therapy (115). The use of esophageal Doppler monitoring to guide fluid administration reduced the rate of postoperative complications in patients undergoing major abdominal surgery compared to fluid therapy guided by conventional parameters (e.g., central venous pressure and arterial pressure) (116).

Similarly to human studies, it has been reported that CO_EDM_ does not present an acceptable agreement with CO_PATD_ in dogs (PE = 39%) (117). Otherwise, an abstract reported a close relationship between stroke distance measured by the esophageal Doppler and SV measured by thermodilution in dogs undergoing hemorrhage and re-transfusion of shed blood (correlation coefficient = 0.9), suggesting that this monitoring device might be a useful surrogate marker of SV changes induced by a fluid challenge (118). Additional studies are necessary to establish the clinical usefulness of the esophageal Doppler as a tool to monitor fluid responsiveness in dogs and cats.

### Electrical Velocimetry

Electrical velocimetry (or transthoracic electrical bioimpedance) is a non-invasive method of continuous CO monitoring based on impedance cardiography. Measurement of left ventricular SV and CO is based on a transcutaneous electrical AC voltage applied to the chest to calculate changes in resistance during the cardiac cycle (119–125). Studies in humans have compared electrical velocimetry with PATD, TPTD, transthoracic, and transesophageal echocardiography with conflicting results (119–125). A recent meta-analysis concluded that electrical velocimetry cannot replace thermodilution or transthoracic echocardiography to measure CO because of an unacceptable precision of agreement (mean PE > 30%) with these methods (126). The clinical utility of electrical velocimetry is also limited in veterinary medicine. In sevoflurane-anesthetized dogs, an electrical velocimetry device showed a marginally acceptable agreement (PE = 30.4%) and a poor ability to track changes in CO_PATD_ (75), which could limit the usefulness of this method to evaluate the response to a fluid challenge in dogs (Table 3).

### Pulse Wave Transit Time

Pulse wave transit time (PWTT) is another recently developed technique that provides continuous, non-invasive CO estimations. This method measures the time elapsed between the R wave of the electrocardiogram, which corresponds to the beginning of ventricular systole, and the appearance of the corresponding pulse wave on a pulse oximeter. Measurement of CO by PWTT is based on the principle that the transit time of pulsatile blood flow, detected by a pulse oximeter, is inversely proportional to CO (76, 93, 127, 128). However, studies have shown that PWTT has an unacceptable agreement with CO_PATD_ in dogs (PE = 61–63%) and humans (PE = 69%) (93, 127). Cardiac output measured by PWTT has also shown a poor agreement (PE = 47%) and poor trending ability (concordance rate = 74%) with CO_TPTD_ (128). In spite of its poor agreement and poor trending ability with reference methods, percent changes in CO measured by PWTT ≥ 11% showed reasonable ability to discriminate the response to a 500 ml fluid challenge (area under the ROC curve = 0.84) during the early phase septic shock humans (93). This observation, and the good ability of PWTT to detect a 15% increase in CO_PATD_ in dogs (76), raises the possibility that this monitoring tool might be useful evaluate fluid responsiveness (Tables 3, 4).

## Controversies of GDFT and the Risk of Fluid Overload

The benefits of volume expansion in hypovolemic individuals cannot be achieved without risks. Although, the physiological principles of GDFT make good sense, evidence of benefit on major outcomes/mortality has been considered inconsistent in the human medical field (6). Otherwise, meta-analysis of 13 clinical trials enrolling 1642 patients, concluded that application of the concept of fluid responsiveness to patients that require acute volume resuscitation appeared to reduce mortality, duration of ICU stay, and duration of mechanical ventilation (3). However, conclusions of this study may be restricted to high-risk surgery, which comprised 12 of 13 trials analyzed (3). While it appears that GDFT in patients undergoing major surgery is beneficial, evidence of an improved outcome of GDFT in septic individuals is lacking (129). In human patients with septic shock there is an association between the volume of fluids administered during resuscitation and the degree of damage to the endothelial glycocalyx, a carbohydrate-rich layer lining vascular endothelial cells that prevents leakage of fluids from the intravascular to the extravascular compartment (130). Theoretically, administering fluids until individuals are at the flat portion of the Frank-Starling curve might further damage the endothelial glycocalyx in septic patients. More recently, dynamic assessment of fluid responsiveness to guide fluid or vasopressor administration in patients with septic shock decreased the total amount of intravenous fluids, lowered the risk of renal and respiratory failure when compared to usual care (45).

### Damage to the Endothelial Glycocalyx and Lung Edema Secondary to Volume Expansion

Repeated fluid challenge administration until the heart is operating on the flat portion of the Frank-Starling curve may potentially lead to tissue/lung edema. Increases in cardiac filling pressures induced by fluid administration stimulates the release of atrial natriuretic peptide by myocardial cells (131), which is a physiological mechanism whereby the organism promotes elimination of excess fluid. However, it has been demonstrated that increased plasma atrial natriuretic peptide also promotes shedding of the endothelial glycocalyx, which may lead to tissue edema (132).

**Figure 6:**
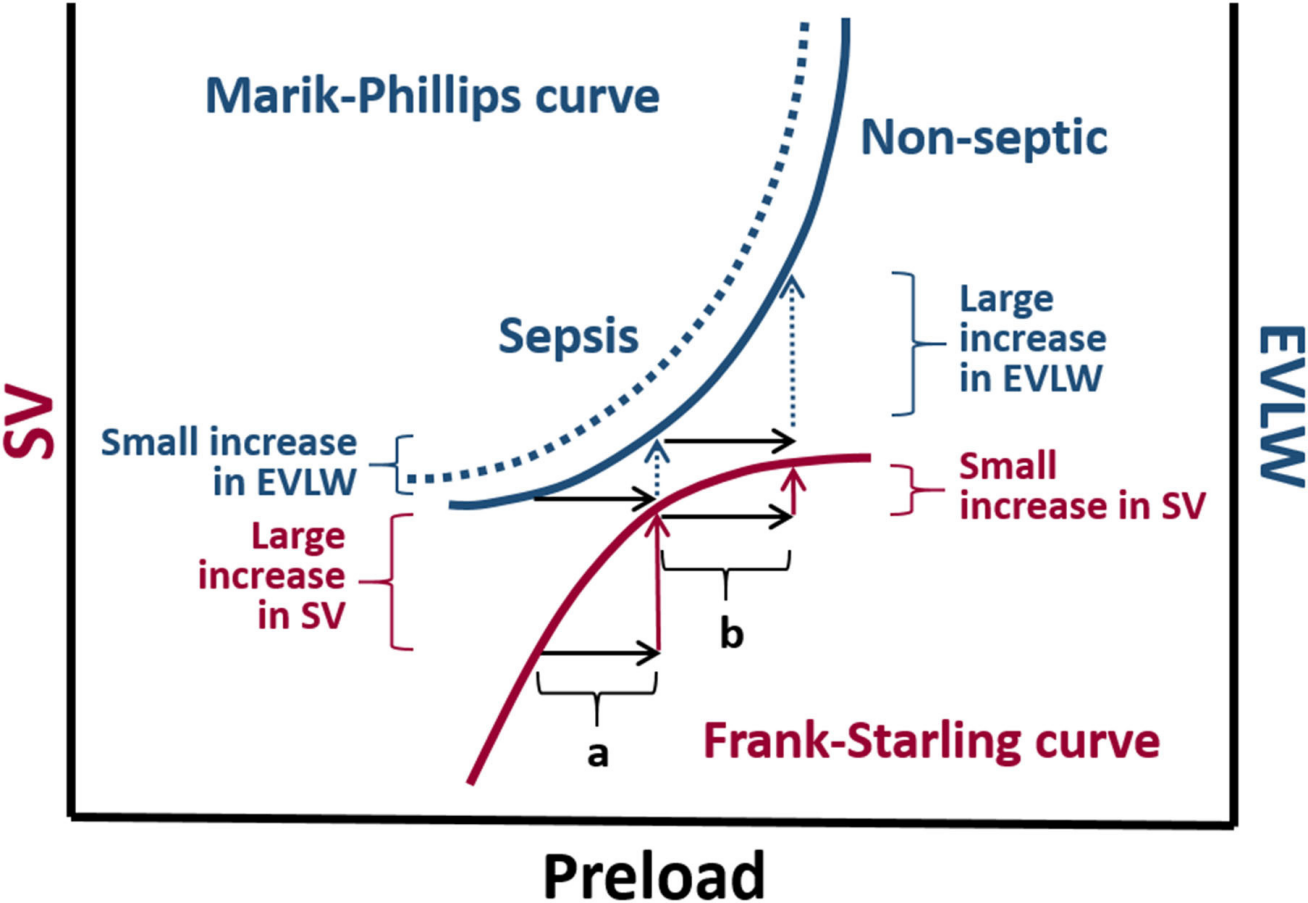
The Marik-Phillips curve and the Frank-Starling curve correlating changes in extra vascular lung water (EVLW) and stroke volume (SV) with preload, respectively. For individuals whose heart is operating on the ascending limb of the Frank-Starling curve, an increase in preload induced by a fluid challenge (a) does not substantially increase EVLW. If a fluid challenge is administered to individuals whose heart is operating on the flat portion of the Frank-Starling curve, the increase in preload (b) may result in a large increase in EVLW. Due to endothelial glycocalyx damage associated with sepsis, larger increases in EVLW can be expected in septic individuals (dashed curve).

Pulmonary edema is a major complication of fluid overload that can be objectively evaluated in human critical care settings by measuring extra-vascular lung water index (EVLWI) and pulmonary vascular permeability index (PVPI) in patients monitored with TPTD technology (133, 134). In human subjects EVLWI > 10 ml/kg and PVPI <2 are suggestive of cardiogenic pulmonary edema; whereas, EVLWI > 10 ml/kg and PVPI > 3 suggests that pulmonary edema is likely caused by increased pulmonary vascular permeability, such as in acute respiratory distress syndrome (133, 134). The superimposition of the Marik-Phillips curve and the Frank-Starling curve, which depict the relationship between EVLWI and SV with preload, respectively, allows understanding why there is an increased the risk of pulmonary edema if the heart is operating near the flat portion of the Frank-Starling curve (135) (Figure 6).

Based on experimental studies healthy anesthetized dogs, it appears that reference values of EVLWI in humans cannot be extrapolated to canine species, as mean EVLWI values of 12 ml/kg have been reported in healthy anesthetized dogs receiving a standard fluid rate of 2 ml/kg/hour of LRS (136). In normovolemic dogs undergoing elective surgery, mean EVLWI values were in the range of 9 to 10 ml/kg and were not significantly increased by one or two fluid challenges with 20 ml/kg of LRS administered over 15 min (16). Although, EVLWI did not increase after volume expansion, an increased incidence of peripheral edema in the form of chemosis and edema of the tongue was recorded the group of animals that were positioned at the flat portion of the Frank-Starling curve after receiving two 20 ml/kg crystalloid boluses (16).

### Use of Point of Care Echocardiography to Evaluate the Risk of Pulmonary Edema Secondary to Volume Expansion

Hydrostatic pulmonary edema may occur if a fluid challenge is administered to individuals presenting a restrictive left ventricular filling pattern. Features of diastolic dysfunction leading to a restrictive filling pattern include an excessive acceleration of early transmitral flow (E wave), and a decreased longitudinal myocardial fiber lengthening at the level of the mitral annulus during early diastole (e' wave), measured by pulse wave Doppler and by tissue Doppler imaging, respectively (137). The E/e' ratio has been in humans used as an estimate of left ventricular filling pressures (LVFP), with values > 14 (e' averaged from the septal and lateral mitral annulus) identifying elevated LVFP with high specificity (low false positive rate) (137).

Myxomatous mitral valve disease and dilated cardiomyopathy are among the heart diseases that lead to elevated LVFP and increased risk of pulmonary edema from bolus fluid administration in canine species (138). In cats, hypertrophic, restrictive, and dilated cardiomyopathy may be associated with elevated LVFP and pulmonary edema (139). The E/e' ratio (e' averaged from the septal and lateral mitral annulus) >12.4 shows a reasonably good ability to discriminate dogs with congestive heart failure (area under the ROC curve = 0.79) (138). However, compared to the E/e' ratio, the ratio between the E wave and the isovolumic relaxation time (IRVT) showed higher accuracy (area under the ROC curve = 0.97) to identify congestive heart failure in canine species (138). In dogs with experimentally induced fluid overload, E/IVRT ratio values > 2.2 are able to identify left atrial pressure values > 15 mmHg with high sensitivity (90%) and specificity (100%) (140). Excessive increases in LVFP due to congestive heart failure in dogs are associated with E/IVRT ratio > values 2.5 and excessively accelerated E waves (>120 cm/s) (138, 141).

Although, data are lacking in veterinary medicine, left ventricular diastolic dysfunction has been recognized as a common feature in humans with sepsis or septic shock (142). Systolic dysfunction (defined as a decrease in left ventricular ejection fraction to <50%) may not be associated with mortality in sepsis. Otherwise, diastolic dysfunction, characterized by a decrease in longitudinal fiber lengthening during early diastole (e') and an increase in E/e' ratio, has been reported to occur more frequently than systolic dysfunction (50 vs. 30% of patients) and has been associated with higher mortality in septic human patients (142, 143). The prevalence of diastolic dysfunction dogs and cats with sepsis/septic shock deserves further investigation as it can impact the prognosis and fluid resuscitation strategies in the emergency setting.

**Figure 7:**
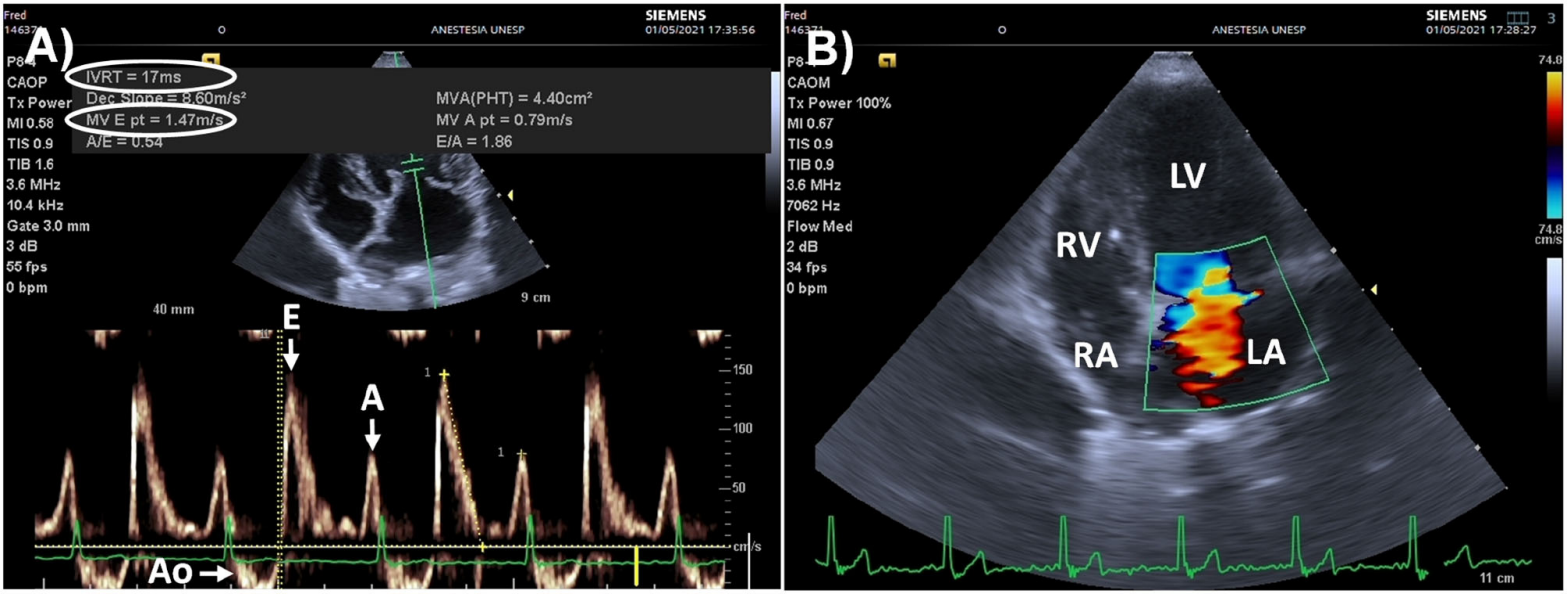
Point of care echocardiography in an 8 kg Schnauzer dog presented for surgery due to a bleeding mass located in the upper lip that presented elevated left ventricular filling pressures (LVFP), secondary to myxomatous mitral valve disease. **(A)** Pulsed wave Doppler of mitral inflow (left apical, four/five-chamber view) showing marked acceleration of the early passive diastolic filling wave (E wave = 147 cm/s, normal range: 53–108 cm/s). The isovolumic relaxation time (IVRT) was low (17 ms, normal range: 43–63 ms), and the E/IVRT ratio was markedly increased (8.64, upper reference value: 2.5), indicating high LVFP due to congestive heart failure. **(B)** Color Doppler (left apical, four-chamber view) showing mitral regurgitation during mid systole. This animal was anesthetized as an emergency procedure with the recommendation for a restrictive fluid therapy strategy. Inotropes/vasopressors instead of fluid boluses were recommended to manage intraoperative hypotension. The congestive heart failure was later managed by a cardiologist. Ao, aorta; E, early passive diastolic filling wave, A, late diastolic filling wave induced by atrial contraction; RV, right ventricle; RA, right atrium; LV, left ventricle; LA, left atrium.

In the presence of echocardiographic changes suggestive of elevated LVFP a restrictive fluid therapy strategy should be considered (Figure 7). Otherwise, recognition of elevated LVFP does not rule out the need for volume replacement. Under these circumstances, the clinician should consider that range of optimal left ventricular filling pressure is relatively narrow and the left ventricle can be easily overfilled (103).

## What is the Ideal Fluid Challenge?

An ideal fluid challenge should reliably discriminate responders from non-responders to volume expansion by means of the smallest volume possible to minimize this risk of fluid overload. An excessively large volume of IV fluids used to test the preload dependency increases the risk of tissue edema. Otherwise, if the volume of fluid used as a “challenge” is excessively small, the resultant increase preload might not be enough to identify responders to volume expansion, yielding false negative results. In hemodynamically stable post-cardiac surgery human patients the administration of progressively increasing volumes of isotonic crystalloids (1 to 4 ml/kg over 5 min) increased the proportion of responders from 20% (1 ml/kg) to 65% (4 ml/kg) (144). Therefore, the ideal fluid challenge is the minimal volume of fluid that still allows identification of all “true” responders to volume expansion.

Not only the volume of fluid, but also the duration of the infusion influences the recognition of responders to volume expansion. Meta-analysis studies in humans have shown that more prolonged infusion times (>30 min) decreases the proportion of responders to volume expansion (145). A third factor that can influence the identification of responders to volume expansion is the timing of the assessment of the change in CO and/or SV. In septic humans, the number of responders to a fluid challenge recorded upon conclusion of a 500 ml crystalloid fluid challenge (individuals where aortic flow VTI increased > 15% from baseline) was decreased by 44% when fluid responsiveness was reassessed 20 min later (111). The increase in preload induced by crystalloid solutions is transient as they rapidly redistribute from the intravascular to the extravascular compartment (146). Persistence of isotonic crystalloids within the intravascular space is likely to be poorer in septic patients because of endothelial glycocalyx damage and low colloid oncotic pressure associated with hypoalbuminemia (130). Meta-analysis studies have not detected an influence of the type of fluid (colloids vs. crystalloids) on the proportion of responders to volume expansion (145). However, fluid challenges with artificial colloids have the potential to induce longer lasting increases in intravascular volume and cardiac preload than similar volumes of isotonic crystalloids.

Experimental evidence suggests that administration of 25% of the “shock dose” of crystalloids in dogs (20 ml/kg) can increase the risk of tissue edema because a larger cumulative volume of fluids may be administered until animals are recognized as non-responders to volume expansion (16). Lower volumes of crystalloids (10 ml/kg over 5 min) may be favored as standard fluid challenge because smaller cumulative volume of fluid is likely to be administered until dogs become non-responders (20). Because longer infusion times of crystalloids result in rapid redistribution of fluid from the intravascular compartment to other compartments (111, 146), infusion times should be kept as short as possible (e.g., 5 to 10 min). The standard crystalloid bolus in cats with signs of shock has traditionally been limited to 10 ml/kg over 15 min because of this species present a smaller circulating blood volume (60 ml/kg of body weight) in comparison to dogs (80 ml/kg of body weight) and because of a perceived higher risk of volume overload in this species. However, clinical data on the use fluid challenges is lacking in cats and crystalloid volumes lower than 10 ml/kg are probably adequate in this species.

## Conclusions

Knowledge on the assessment of fluid responsiveness has experienced a significant development during the past two decades in the human medical field. In patients admitted with signs of circulatory failure, fluid resuscitation guided by a fluid challenge approach has the potential to improve patient outcome. Considering the principle of “less is more,” to minimize the risk of fluid overload, it has been recognized that smaller volumes of fluids, or mini-fluid challenges, may allow identification of responders to volume expansion induced by a standard fluid challenge. To reduce the risk of edema from fluid overload, recommendations for the use of 25% of the “shock dose” should be revised in favor of smaller volumes of crystalloids as standard fluid challenge (up to 10 ml/kg over 5–10 min in dogs and <10 ml/kg over 10–15 min in cats). When using the fluid challenge approach, it should be recognized that the response variable measured before and after the fluid challenge (i.e., the method of CO and SV measurement or its surrogates) will significantly impact the identification of responders to volume expansion. In veterinary medicine, goal-directed fluid therapy aiming to maximize CO and SV is in its infancy. Among the CO and SV measurement technologies that could be used to assess the response to a fluid challenge, aortic flow VTI obtained by transesophageal and transthoracic echocardiography appears to be a promising tool to evaluate fluid responsiveness in animals. Esophageal Doppler monitoring might be particularly useful to guide fluid administration in anesthetized dogs and cats or in animals under mechanical ventilation in the ICU. Future studies are needed to assess fluid responsiveness status in dogs and cats with signs of circulatory failure and the potential benefits that could be achieved by maximizing CO and SV *via* fluid administration in high-risk small animal patients.

## Author Contributions

FT-N: manuscript conception and writing (~70% of manuscript text). AV: Manuscript preparation, writing (~30% of manuscript text), and critical review. Both authors contributed to the article and approved the submitted version.

## Conflict of Interest

The authors declare that the research was conducted in the absence of any commercial or financial relationships that could be construed as a potential conflict of interest.

## Publisher's Note

All claims expressed in this article are solely those of the authors and do not necessarily represent those of their affiliated organizations, or those of the publisher, the editors and the reviewers. Any product that may be evaluated in this article, or claim that may be made by its manufacturer, is not guaranteed or endorsed by the publisher.
